# Active repurposing of drug candidates for melanoma based on GWAS, PheWAS and a wide range of omics data

**DOI:** 10.1186/s10020-019-0098-x

**Published:** 2019-06-20

**Authors:** Ali Khosravi, B. Jayaram, Bahram Goliaei, Ali Masoudi-Nejad

**Affiliations:** 1 Laboratory of Systems Biology and Bioinformatics (LBB), Institute of Biochemistry and Biophysics, University of Tehran, Tehran, Iran; 2 Department of Chemistry & Supercomputing Facility for Bioinformatics & Computational Biology, Kusuma School of Biological Sciences, Indian Institute of Technology, Delhi, India; 3 Department of Biophysics & Bioinformatics, Institute of Biochemistry and Biophysics (IBB), University of Tehran, Tehran, Iran

**Keywords:** Drug repurposing, Melanoma, GWAS, PheWAS, Transcriptomic, Metabolomics, Medical dermatology, Oncology, Drug response

## Abstract

**Background:**

Drug repurposing is a swift, safe, and cheap drug discovery method. Melanoma disorders present low survival and high mortality rates and are challenging to diagnose and treat. Moreover, there is a high volume of worldwide investigations that are attempting to find melanoma-related genes of influence, which can be identified as responsive targets for reliable treatment.

**Method:**

In this study, we used a wide range of data analyses to analyze over 1100 genes and proteins of influence with respect to cutaneous malignant melanoma. Our analysis included various investigational results from genome- and phenome-wide association studies (GWAS and PheWAS, respectively), biomedical, transcriptomic, and metabolomic datasets. We then researched the DrugBank for potential melanoma targets from the selected list. We excluded known melanoma targets to obtain a list of druggable proteins. We performed a precise analysis of the drugs’ pathogenesis and checked the expression profiles of the selected drugs having high associations with known anti-melanoma drugs.

**Result:**

We found 35 drugs that interacted with 20 unique targets. These drugs appear to have high melanoma treatment potentials. We confirmed our results with previous studies and found supporting references for 30 of these drugs.

In conclusion, this investigation can be applied to various diseases for the efficient and economical repurposing of various drug compounds. For further validation, the results may be applicable for in vivo tests and clinical trials.

## Background

Cutaneous malignant melanoma is one of the most perilous diseases in the world, affecting more than millions of people globally. According to world health organization, there are over 2 to 3 million cutaneous malignant melanoma diagnoses every year. Recent reports from the American Cancer Society revealed that the five-year survival rate for patients with early stage detection of skin cancer is approximately 99% in the U.S. Cutaneous malignant melanoma has excessive morbidity and mortality rates (Cummins et al. 2006). The survival time can drop to 63% when the disease affects lymph nodes, and it can lower to 20% in regard to metastasize other organs (Balch et al. 2009).

Disease etiology is the most important step towards finding a feasible treatment for any disease. According to reports that were published earlier, melanoma is mainly due to the influence of the UV-damage response to genomic content in humans. Genomic instability caused by UV-radiation and other factors have various resources (Parkin et al. 2011), including the influence of the tumor suppressor gene, cell cycle inhibitors, oncogene activities, molecular and cytogenetic changes and telomere dysfunction, and others (Elder 1999). Although these resources are mainly induced by internal genomic content, according to the genetic interpretation of the population, environmental factors are key operating agents for such diseases (Han et al. 2005).

Understanding the disease etiology can highlight the inducing genes, which are directly or indirectly affected by the progress of cancer. In this study, we collected influencing gene from multiple sources, with strong biological evidence. Genome-wide association studies (GWAS) (MacArthur et al. 2017), phenome-wide association studies (PheWAS) (Denny et al. 2013), transcriptome analysis (Berger et al. 2010), metabolomics analysis (Wishart et al. 2018a) and disease-gene association studies (Babbi et al. 2017) are used to reveal these associated genes.

Apart from finding the genes that are associated with melanoma, it is highly important to find a therapeutic solution to reduce the number of patients who are suffering. Drug repurposing (or drug repositioning), which has become a conventional drug development procedure, can reuse already available, well-studied, marketed drugs for new indications (Li et al. 2016). Drug repurposing can reduce the time for identification of the leading compound for 3–12 years; it can also reduce the risk and lower the cost of drug development (Ashburn and Thor 2004). In this study, we used the “Disease Based Approach” as one of multiple drug-repurposing approaches (Sun et al. 2017; Zhang et al. 2015; Li et al. 2016). Hence, the leading compound will be identified by an analysis of target-based interactions with the associated genes corresponding to a well-known disease.

In this study, systematic approaches are involved in finding drug compounds for melanoma treatment. We have approached advanced biological technology, such as GWAS, PheWAS, expression profiles, and biomedical and metabolomics data, to obtain the highest operational druggable biomarker and their corresponding leading molecule from already available drugs. We finally used the text-mining approach to validate our candidate drug compound from previous studies.

## Materials and methods

### Dataset collection

To obtain the dataset for skin cancer-related genes, proteins and metabolites, we used several databases and catalogs. In this study, we used genomics, transcriptomics and metabolomics, and biomedical data from various sources, which is described as follows.

#### Genome wide association studies

We have revealed all associated genes for melanoma disorders from the GWAS catalog (MacArthur et al. 2017). We have collected information on 1) associated genes; the nearest gene associated with an allele; 2) SNPs, the unique SNP number; 3) patient ethnicity, the ethnicity of population study; 4) publication information, PubMed ID; 5) *P*-value, the *p*-value of the allelic association to the disorder; and 6) the phenotypic trait from the catalog; associated disorder. The selection of the disease/trait attribute as “melanoma” and “cutaneous malignant melanoma” has been done in this process.

#### Phenome wide association studies

Utilization of phenome-wide association studies (PheWAS) for drug repurposing has been done successfully during past years (Rastegar-Mojarad et al. 2015; Yin et al. 2018; Khosravi et al. 2019). In this study, we have used the PheWAS catalog to discover all associated genes corresponding to variant alleles for different ethnicities around the world (Denny et al. 2013). The comorbidity of different phenotypic traits has been traced by variation in allelic content due to a shared biological mechanism and/or environmental effect, such as UV radiations (Bush et al. 2016). We have retrieved the information from the catalog by setting the phenotype as “melanoma” and “skin cancer” and extracted the following data: 1) SNPs, SNP accession number; 2) PheWAS phenotype, the associated disorder; 3) *p*-value, the p-value corresponding to the association of SNPs and disorders; and 4) gene name; nearest gene associated with an allele.

#### Metabolomics data

Metabolomics analysis is used significantly as a drug discovery methodology. The analysis highlights preclinical research and biomarker detection. Cancer-related metabolites are associated with enzymes and transporters as the melanoma biomarkers are leading targets for a drug-repurposing methodology (Robertson and Frevert 2013). It can be used as patient stratification and help translational medicine discovery (Srivastava and Creek 2018). In this study, we have used the Human Metabolome Database (HMDB) (Wishart et al. 2018a) for mining melanoma-related proteins. We retrieved the information by exploring all the metabolites that are associated with melanoma disorder by searching “melanoma” and “malignant melanoma” from the database. In next step, we manually collected the proteins that are associated with the respected metabolites.

#### Transcriptome data

RNA-Seq and transcriptomics analysis can improve the productivity of biomedical research to obtain more precise compounds in the drug discovery process (Zhao et al. 2014). As genomic mutation and their corresponding epigenetic changes can alter gene expression and functions, the study of DNA microarrays has great importance (Atak et al. 2013). Misplacement in the DNA copy number can be dignified by the DNA microarray and can also alter gene expression profiles (Kumar-Sinha et al. 2015). The collected dataset was in terms of differentially co-expressed genes that are affected by melanoma disorder (Berger et al. 2010). The study provided differentially co-expressed gene names as leading targets for the identification of anti-melanoma drug compounds.

#### Biomedical data

A modern biomedical technique has enhanced genetic research by discovering different genetic component of phenotypic traits. We have used eDGAR database (Babbi et al. 2017) to reveal skin cancer-associated genes with annotated relationships among them. eDGAR collected disease-based associated genes deposited in OMIM (Amberger et al. 2015), UniProt (UniProt Consortium 2015) and CLINVAR (Landrum et al. 2016) databases. These genes were revealed from the phenotype OMIM ID 155600 corresponding to cutaneous malignant melanoma.

### Metabolites protein network reconstruction

We reconstructed the metabolites enzymes/transporters networks by mapping their interconnections derived from metabolomics data. We prepared a flat-file, which states metabolites and their associated protein and the type to reconstruct the network. As you can see in the result section 3.2, we visualized the network with the help of Cytoscape tools (Shannon et al. 2003).

### Drug mapping

**Fig. 1:**
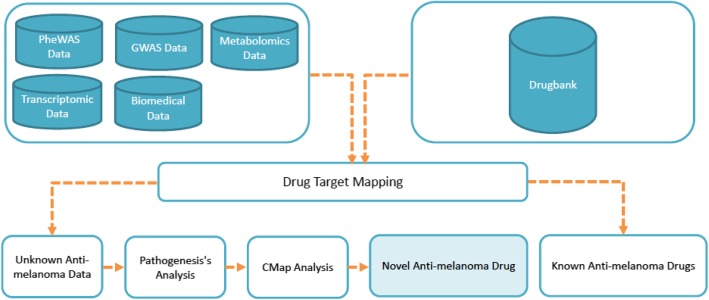
Drug repurposing approach to identify novel anti-melanoma drugs; in this approach we have collected melanoma responsive genes/proteins and identified target genes by drug target mapping with the help of drugbank. We have removed all known anti-melanoma drugs and analyzed the unknown anti-melanoma drugs by pathogenesis information and CMap analysis. We have found 35 drugs interacting with 20 targets

We collected all melanoma-associated genes and proteins from GWAS, PheWAS, transcriptomics, metabolomics and biomedical studies as described above. The method has been shown in Fig. 1. This set contains more than 1100 associated genes and proteins having the maximum influence on the disorder. You can find the dataset in supplementary file 1 (https://github.com/LBBSoft/Melanoma). We tracked different drug-related databases and found DrugBank 5.0 (Wishart et al. 2018b) to be one of the best updated databases containing approximately 17,000 drug-target associations and information on over 10,000 drug compounds. We have mapped the drug-target information with our set of genes and proteins in the DrugBank database. We have revealed the drug name, mode of action, target name, current indications and drug groups from the DrugBank. We also excluded all experimental, illicit, withdrawn and investigational molecules from our data and only considered the approved drug-target information in this analysis. We have excluded 638 unique drugs interacting with 165 target genes, which are available in supplementary file 2 (https://github.com/LBBSoft/Melanoma).

### Pathogenesis information validation

We have analyzed the pathogenic nature of the revealed drug molecules to comprehend drug antagonists or agonists (Wang and Zhang 2013). We revealed anti-melanoma pathogenesis information through biomedical records and literature reviews from PubMed central. We analyzed the dataset for pathogenetic information of genes, such as the gain of function (GOF) and loss of function (LOF) roles in humans for anti-melanoma efficacy. We used the OMIM (Amberger et al. 2015) database mainly to obtain this information. As you can see in the results section 3.5, we removed the genes/proteins with suitable pathogenetic information with respect to the transporter action of the drug and maintained genes/proteins with reliable scientific reports.

### Connectivity map analysis

The connectivity map (CMap) is a drug-response expression profile analysis on various human-cultured cell lines. This approach shows a transcriptional expression profile on the treatment of drugs on human-cultured cell lines (Lamb et al. 2006). CMap qualifies the drug target associations that are correlated to melanoma through gene expression profiles. We have analyzed all drug candidates from the above states in CMap to comprehend their mechanisms of action and anti-cancer effects.

## Results

### Systematic collection of melanoma-related biomarkers

As described above, the melanoma-related GWAS dataset consists of 55 unique genes mapped by different SNP-risk alleles. The minimum and maximum *p*-value of collected data are 4E-37 and 8E-06, which are used for high demand and an accurate analysis. The *P*-value in GWAS and PheWAS studies is a probability of the type I error that is made in hypothesis testing. The P-value signifies the possibility of randomness in finding any disease associated to a specific variant. These data have been compiled from 9 different studies. Similarly, we have revealed 765 alleles by looking for “melanoma and skin cancer” keywords in the PheWAS catalog. These SNPs correspond to 260 unique associated genes in our analysis. The *p*-value of the revealed genes is in the range of 0.05 to 1.106E-16.

**Fig. 2:**
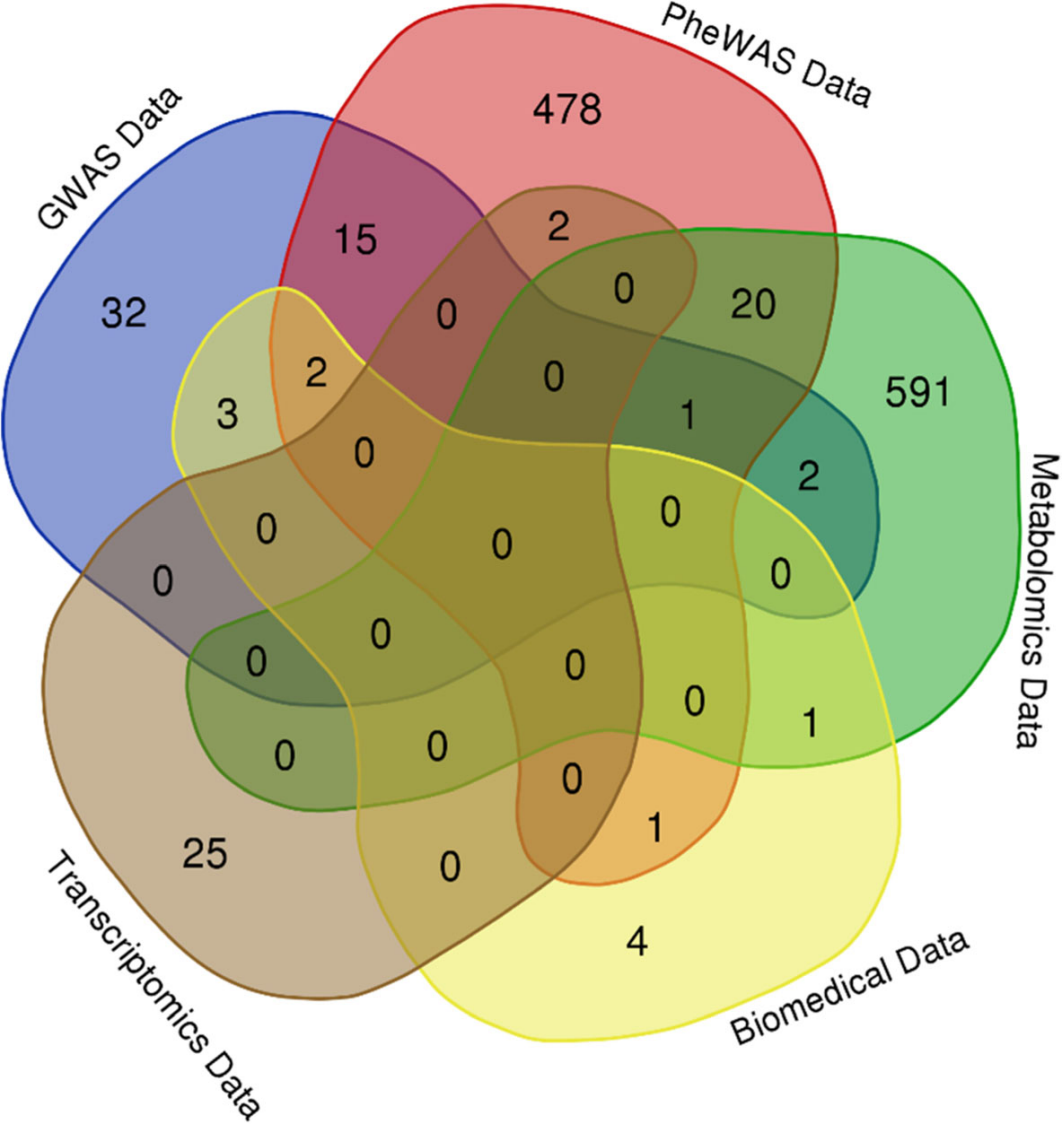
Venn diagram showing the logical distribution of melanoma associated genes and proteins from revealed from various approaches. Here we have five different datasets for melanoma responsive genes or proteins. There are 50 melanoma associated genes or proteins discovered from more than one approach showing high accuracy in disease related target identification

We exposed 23 metabolites that are associated with 616 unique proteins in the metabolomics dataset with significant actions in the form of enzymes, transporters and unknowns in melanoma. In addition, we included 27 validated differentially expressed genes from the melanoma transcriptome. Finally, we incorporated 11 unique genes that are associated with cutaneous malignant melanoma from biomedical databases. The set of genes and proteins has 1178 unique members. These members have been identified and were discovered from different approaches, and some of them have been confirmed from more than one approach. The distribution of genes is available in Fig. 2.

### Metabolites protein network analysis

**Fig. 3:**
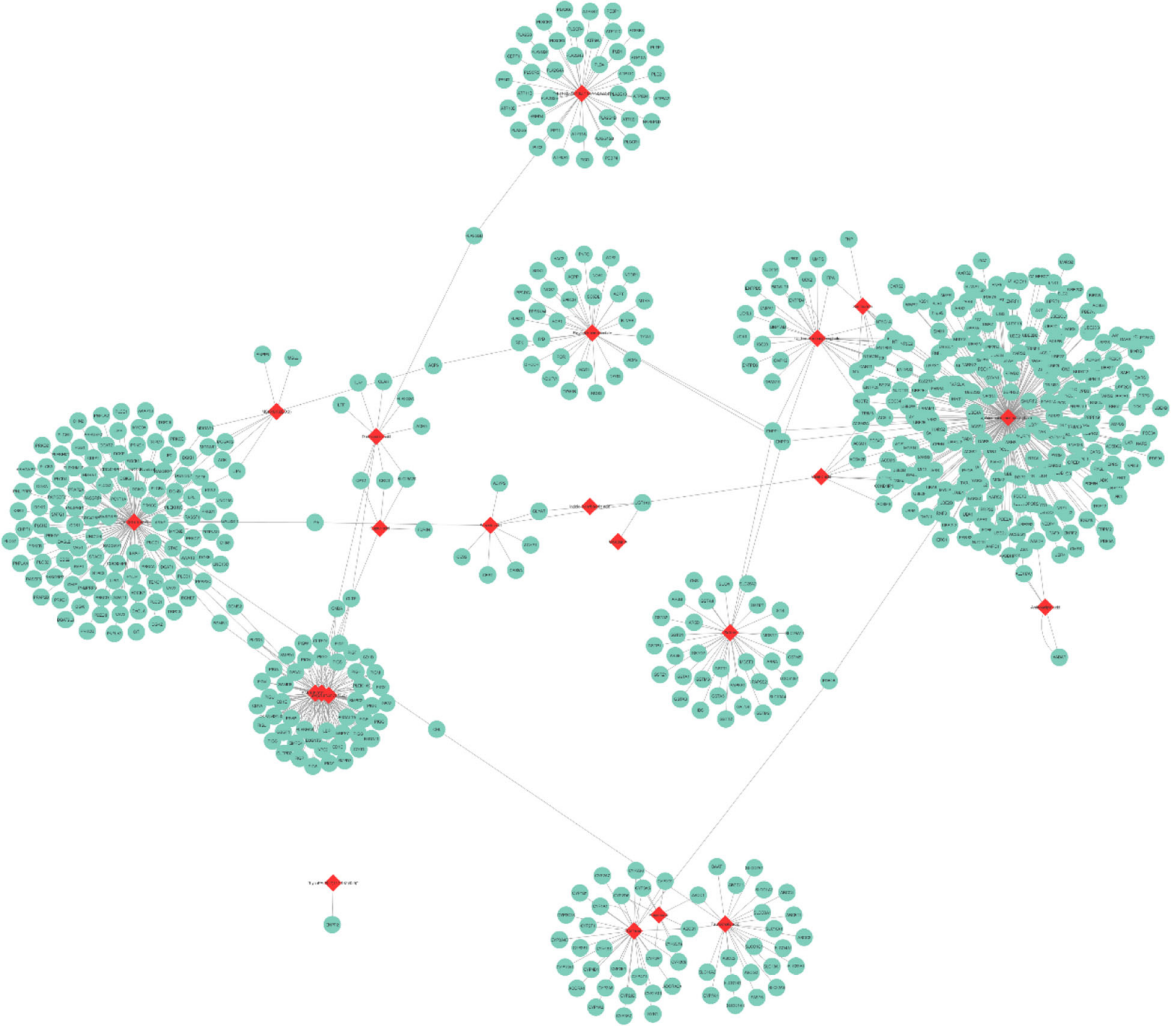
Metabolites-protein network reconstruction for cutaneous malignant melanoma; red node represent metabolites and green nodes signify proteins in the form of enzymes and transporters and the edges shows their common association which has been revealed from HMDB. There are 23 unique metabolites connecting to 617 associated enzymes or transporters

We found approximately 800 metabolite-protein associations related to melanoma disorders in the dataset. The proteins in the dataset were in the form of enzymes, transporters or sometimes unknown. To rely on the dependability of our finding, we reconstructed a protein-metabolite network consisting of proteins/metabolites as nodes and their associations as edges. This highly connected graph, which is shown in Fig. 3, was visualized by Cytoscape (Shannon et al. 2003). There are 23 unique metabolites connecting to 617 associated enzymes or transporters.

### Drug-target mapping

All melanoma risk biomarkers, which consist of a set of genes and proteins corresponding to melanoma disorder, are gathered here. We have four types of targets in the form of protein (215 drugs associated with 127 targets), enzyme (604 drugs associated with 56 targets), transporter (362 drugs associated with 33 targets) and carrier (6 drugs associated with 4 targets). We have mapped all types of targets with the melanoma risk biomarkers to find druggable targets. Hence, by analyzing over 1170 genes, we left with 193 mapping targets. We found 731 potential drug molecules in this step, which are available in supplementary file 3 (https://github.com/LBBSoft/Melanoma). We retrieved the drug name, transporter action, target name, indication, FDA label and DrugBank ID.

### Unknown melanoma potential drug

We eliminated the drug molecules with melanoma indications from our proposed drugs. There are 215 melanoma-related drug compounds in phase 0 to 4 with different statuses, such as completed, recruited, suspended, withdrawn, not yet recruited and terminated. We found 75 common drug compounds in both melanoma-related drugs and in potential drug compounds. By eliminating common drug lists from our potential drug compound, we left with 658 drug molecules associated with 184 targets.

### Melanoma drug pathogenesis analysis

We used OMIM (Amberger et al. 2015) and other scientific reports to find pathogenic information on various targets. As the pathogenesis is mainly correlated to genetic disorders, we have looked to find pathogenic information on the melanoma disorder. We considered the pathogenic information of 74 targets associated with 361 drug compounds.

We have analyzed the effect of the pathogenic gene function (loss and gain of function) to drug-targets looking at the transporter actions (agonist and antagonist) retrieved for each candidate drugs from the DrugBank. By removing nonmatching genes, whose activities do not suit their pathogenetic information, we obtained 277 potential drugs interacting with 74 targets. The related data in this step is available in supplementary file 4 (https://github.com/LBBSoft/Melanoma).

### Connectivity map analysis

We evaluated the negative association of the “selegiline” drug, which inhibits the apoptosis in M-1 human melanoma cells and the positive association of anti-melanoma-related drugs such as “paclitaxel” and “vinblastine” to obtain a suitable expressional effect on the treatment of the melanoma disorder. We revealed the drug expression profile by looking for different drugs based on a signature query in CMap. As CMap covers the multiple drug expression in human cell lines, it will pick the most expressed drug in the available human cell line. Hence, we have achieved our final anti-melanoma potential drugs by utilizing the correlation of known anti-melanoma and proposed drugs with the help of the mean and enrichment score. Many of the drug compounds did not have a positive correlation with known anti-melanoma drugs, and some were not found in the CMap database. We left with 35 drug compounds that are associated with melanoma therapy. These drug compounds correspond to 20 targets of the protein, enzyme and transporter types. The final repurposed drug candidates are shown in Table 1.

**Table 1:** Anti-melanoma repurposed candidate drug molecules and supporting information based on a wide range of data analyses

Drug Name	Current drug indication	Stage	Target	Action mode	Pathogenesis	Supporting evidence
Erythromycin	Respiratory tract infections	Recruiting	CYP3A7	Inhibitor	GOF, human drug metabolizing enzyme (Neunzig et al. 2011)	NA
Milrinone	Congestive heart failure	Recruiting	PDE3A	Inhibitor	GOF, Targeting tumor cells (Nazir et al. 2017)	(Murata et al. 2002)
Dapsone	Leprosy and dermatitis herpetiformis	Completed	CYP3A7, NAT2	Substrate	LOF, Arylamine n-acetyltransferase activity^#^	(Kyllo et al. 2014)
Theophylline	Chronic asthma	NA	CYP1A2, CYP1B1, CYP2D6, PDE3A	Inhibitor, Substrate	GOF, Phosphodiesterase Inhibitors^#^	(Steinberg and Whittaker 1976; Wick 1981)
Gefitinib	Metastatic nonsmall cell lung cancer	Completed	CYP1A1, EGFR	Antagonist	GOF, Ubiquitin protein ligase binding^#^	(Djerf et al. 2009)
Omeprazole	Duodenal ulcers	Completed	ABCC3, CYP1A1, CYP1A2	Inducer	LOF, Organic anion transmembrane transporter activity^#^	(Matsui et al. 2015)
Levonorgestrel	Enopausal and postmenopausal disorders	Completed	CYP19A1, ESR1	Inhibitor	GOF, Nuclear hormone receptor (Grostern et al. 2001)	(Kjaeldgaard et al. 1988)
Mexiletine	Ventricular fibrillation	Completed	CYP1A2	Inhibitor	LOF, Oxidoreductase activity (Kuraishi et al. 2003)	(Kuraishi et al. 2003; Andoh et al. 2008)
Rosiglitazone	Type 2 diabetes mellitus	Completed	CYP2A6	Inhibitor	GOF, Steroid hydroxylase activity^#^	(Mossner et al. 2002)
Chloramphenicol	Etracycline-resistant vibrios	Recruiting	CYP3A7	Inhibitor	GOF, Oxygen binding^#^	(Lamb et al. 2015)
Buspirone	Anxiety disorders	Completed	CYP3A7	Substrate	LOF, Oxygen binding^#^	NA
Zidovudine	Human immunovirus infections	Completed	CYP2A6, TERT	Substrate, Inhibitor	GOF, Steroid hydroxylase activity (Hahn et al. 1999)	(Fang and Beland 2009)
Flutamide	Prostate cancer	Completed	CYP1B1	Substrate, Inhibitor	GOF, Oxygen binding^#^	(Hsueh et al. 2003)
Cimetidine	Peptic ulcer disease	Completed	SLC22A5, CYP3A7	Inhibitor	GOF, Symporter activity (Flodgren et al. 1983)	(Flodgren et al. 1983; Harland and Saihan 1989)
Diclofenac	Osteoarthritis and rheumatoid arthritis	Completed	PLA2G2A	Inhibitor	GOF, Phospholipid binding^#^	(Albano et al. 2013)
Monobenzone	Skin vitiligo	NA	TYR	Inhibitor	GOF, Protein homodimerization activity (Chen et al. 2009)	(van den Boorn et al. 2010)
Trazodone	Depression	Completed	CYP3A7	Substrate	LOF, Oxygen binding^#^	(Chang and Lin 2011)
Verapamil	Hypertension, angina, and cluster headache	Completed	SLC22A5	Inhibitor	GOF, Symporter activity^#^	(Robinson et al. 1986; Formelli et al. 1988)
Cefixime	Various infections	Completed	SLC22A5	Inhibitor	GOF, Symporter activity (Ganapathy et al. 2000)	NA
Flurbiprofen	Osteoarthritis and ankylosing spondylitis	Completed	UGT1A1	Inhibitor	GOF, Steroid binding (Zhou et al. 2009)	NA
Norethisterone	Dysfunctional Uterine Bleeding	Completed	CYP3A7	Substrate	LOF, Oxygen binding (Preissner et al. 2010)	(Kjaeldgaard et al. 1988)
Risperidone	Schizophrenic disorders	Completed	CYP3A7	Substrate	LOF, Oxygen binding (Preissner et al. 2010)	(Uzawa et al. 2014)
Hydrocortisone	Acute Gouty Arthritis	Completed	CYP3A7	Substrate	LOF, Oxygen binding (Preissner et al. 2010)	(Wang et al. 2018; Rathore et al. 2016)
Estradiol	Vasomotor symptoms	Completed	CYP1B1, CYP2C8, CYP3A7, UGT1A1, ESR1	Agonist	LOF, Zinc ion binding^#^	(Kanda and Watanabe 2001; Poletini et al. 2016; Li et al. 2017)
Tacrolimus	Heart Transplant Rejection	Completed	CYP3A7	Substrate	LOF, Oxygen binding^#^	(Matsumoto et al. 2018; Puza et al. 2018)
Zalcitabine	Human Immunodeficiency Virus	Completed	DCK	Substrate	GOF, Protein homodimerization activity (Rossi et al. 1999)	(Hardeman et al. 2017)
Acetylsalicylic acid	Moderate Pain	Completed	EDNRA	Inhibitor	GOF, Receptor for endothelin-1^#^	(Kumar et al. 2018)
Rifampicin	Tuberculosis	Completed	ABCC3, CYP2A6	Inducer, inhibitor	GOF, Steroid hydroxylase activity^#^	(Levavasseur et al. 2016)
Praziquantel	Schistosoma infection	Completed	CYP3A7	Substrate	LOF, Oxygen binding^#^	NA
Norfloxacin	Urinary tract infection	Recruiting	SLC22A5, CYP3A7	Inhibitor	GOF, Symporter activity^#^	(Gouvea et al. 2012)
Amiodarone	Recurrent ventricular fibrillation	Completed	CYP2A6, CYP3A7	Inhibitor	GOF, Steroid hydroxylase activity (Zhou et al. 2009)	(Zuba et al. 2016)
Bupropion	Depression, Bipolar	Completed	CYP2A6	Substrate	LOF, Steroid hydroxylase activity^#^	(Ashrafi et al. 2018)
Mitoxantrone	Progressive relapsing	Completed	CYP1B1	Inhibitor	GOF, Oxygen binding^#^	(Yu et al. 2015; Yu et al. 2016)
Saquinavir	Human Immunodeficiency Virus	Completed	CYP3A7	Substrate	LOF, Oxygen binding^#^	(Mijatovic et al. 2011; Donia et al. 2012)
Clomipramine	Obsessive Compulsive Disorder	Completed	GSTP1	Inhibitor	GOF, S-nitrosoglutathione binding^#^	(Parker et al. 2012)

^#^The information provided by OMIM database

These drugs have an enrichment score in the range of − 0.167 to − 0.755. The *p*-value and specificity of most of the repurposed drug were not available. The CMap detail of the repurposed drug is available in supplementary file 5 (https://github.com/LBBSoft/Melanoma).

## Discussion

In this study, we used a wide range of data analyses to find the responsive genetic content to reveal potential melanoma-related targets. These targeting genes and proteins have associated drug compounds that are approved but have not been reported as melanoma-treating drugs. We found 35 drug candidates interacting with our analyzed set of targeted genes and proteins. These drugs are of different human pathogeneses and action modes, which have a treatment efficacy over melanoma patients. Apart from that, based on the analysis of the connectivity map we found, our candidate drugs have a positive association of melanoma known drugs expression profile over human cell lines.

As is shown in Table 1, we found supporting biological evidence for the applicability of 30 drug compounds as potential drug candidates for the treatment of melanoma in previous scientific reports. These reports concentrated on finding a feasible effect of drug candidates in melanoma or skin cancer cell lines or various other animal tests. There are only 5 drug candidates, which have no related previous studies showing their anti-melanoma effects.

The repurposed drug candidates are highly effective in the inhibition of cell proliferation in melanoma cells. The induction of apoptosis in melanoma cells is also one of major results of the drug’s effects on the disease. There are other reports stating the effect of various drugs in the alteration of the melanoma cellular function, which can include various metabolic functions that alter the energy level of metabolites in melanoma cells and their effect on mitochondrial and other pathways.

We used the most precise target identification approaches to select melanoma-related genes and proteins. These approaches include all types of genetic analyses, including genetic variation, expression profiling, biomedical associations and metabolomics pathways. Target level analyses of given genes and proteins show that the resulting candidate drug targets are from 20 unique genes and proteins. These genes have been selected from a various wide range of data analyses. The revealed genes and protein distributions are as follows: 6 targets from PheWAS data, 3 targets from GWAS data, 14 targets from metabolomics data and 2 targets from biomedical data and no selected targets from transcriptomics data. The list of gene/protein names followed by the method of analysis is given in supplementary file 6 (https://github.com/LBBSoft/Melanoma).

We have seen the TYR, TERT, CYP1B1, CYP19A1 and CYP1A1 genes/proteins, which are the candidate drug targets fetched by more than one method of analysis. TYR is revealed in PheWAS, GWAS and biomedical datasets. TERT is revealed in GWAS and biomedical datasets. CYP1B1 is found in the metabolomics and GWAS dataset. Finally, CYP19A1 and CYP1A1 are found in both the metabolomic and PheWAS datasets.

The connectivity map (CMap) analysis includes repurposed drugs with high positive (paclitaxel and vinblastine) or negative (selegiline) correlations of mean and enrichment scores; hence, only drugs with a similar mechanism of action will be included in this approach. However, this method may bypass some anti-melanoma potential drugs with a novel mechanism of action, which can be noted as the limitation of this approach.

This in silico approach can be used for various disorders and has a high potential for nominating sets of novel repurposed drugs with higher performance. This approach used a wide range of data analyses with the help of various datasets to invoke responsive genes and proteins. As databases do not cover only curated data and contains predictive and putative data, databases may contain several false-positive or false-negative data. Apart from that, an analysis of CMap did not consider the expression of drugs on melanoma-specific cell lines and may have few errors. Therefore, in vitro or in vivo experiments and, later, animal and clinical trials are required to repurpose these candidate drugs.

## Conclusions

Cutaneous malignant melanoma is a highly dangerous disorder that has high mortality and less survival time. Due to the non-availability of treatment drugs, treating the disease is costly and painful. We have used a wide range of analyses to reveal the melanoma-related influencing genes and found corresponding druggable proteins. In this methodology, we excluded known melanoma drugs and their respected targets from the dataset. The pathogenesis information of selected targets has been analyzed based on the disorder and pharmaceutical actions. Furthermore, the resulting drug targets have been analyzed based on the expression profile of drugs to the human cell line with the help of the CMap Tool. We found 35 potential drugs interacting with 20 targets, which can treat melanoma disorder. This approach can be used to find potential treatment drugs for other disorders.

## Data Availability

The datasets generated and/or analyzed during the current study are available from the corresponding author upon reasonable request.

## Abbreviations

CMap: Connectivity map
FDA: Food and Drug Administration
GOF: Gain of function
GWAS: Genome Wide Association Studies
HMDB: Human Metabolome Database
LOF: Loss of function
OMIM: Online Mendelian Inheritance in man
PheWAS: Phenome Wide Association Studies
SNP: Single Nucleotide Polymorphism
UniProt: Universal Protein resource
UV: Ultraviolet
